# Diagnostic signature for heart failure with preserved ejection fraction (HFpEF): a machine learning approach using multi-modality electronic health record data

**DOI:** 10.1186/s12872-022-03005-w

**Published:** 2022-12-26

**Authors:** Nazli Farajidavar, Kevin O’Gallagher, Daniel Bean, Adam Nabeebaccus, Rosita Zakeri, Daniel Bromage, Zeljko Kraljevic, James T. H. Teo, Richard J. Dobson, Ajay M. Shah

**Affiliations:** 1 King’s College London British Heart Foundation Centre of Excellence, School of Cardiovascular and Metabolic Medicine and Sciences, King’s College London, James Black Centre, 125 Coldharbour Lane, London, SE5 9NU UK; 2 Department of Biostatistics and Health Informatics, Institute of Psychiatry, Psychology and Neuroscience, King’s College London, London, UK; 3 Health Data Research UK London, Institute of Health Informatics, University College London, London, UK; 4 King’s College Hospital NHS Foundation Trust, London, UK; 5 NIHR Biomedical Research Centre at South London and Maudsley NHS Foundation Trust and King’s College London, London, UK

**Keywords:** HFpEF, Machine learning, Dyspnea

## Abstract

**Background:**

Heart failure with preserved ejection fraction (HFpEF) is thought to be highly prevalent yet remains underdiagnosed. Evidence-based treatments are available that increase quality of life and decrease hospitalization. We sought to develop a data-driven diagnostic model to predict from electronic health records (EHR) the likelihood of HFpEF among patients with unexplained dyspnea and preserved left ventricular EF.

**Methods and results:**

The derivation cohort comprised patients with dyspnea and echocardiography results. Structured and unstructured data were extracted using an automated informatics pipeline. Patients were retrospectively diagnosed as HFpEF (cases), non-HF (control cohort I), or HF with reduced EF (HFrEF; control cohort II). The ability of clinical parameters and investigations to discriminate cases from controls was evaluated by extreme gradient boosting. A likelihood scoring system was developed and validated in a separate test cohort. The derivation cohort included 1585 consecutive patients: 133 cases of HFpEF (9%), 194 non-HF cases (Control cohort I) and 1258 HFrEF cases (Control cohort II). Two HFpEF diagnostic signatures were derived, comprising symptoms, diagnoses and investigation results. A final prediction model was generated based on the averaged likelihood scores from these two models. In a validation cohort consisting of 269 consecutive patients [with 66 HFpEF cases (24.5%)], the diagnostic power of detecting HFpEF had an AUROC of 90% (P < 0.001) and average precision of 74%.

**Conclusion:**

This diagnostic signature enables discrimination of HFpEF from non-cardiac dyspnea or HFrEF from EHR and can assist in the diagnostic evaluation in patients with unexplained dyspnea. This approach will enable identification of HFpEF patients who may then benefit from new evidence-based therapies.

**Supplementary Information:**

The online version contains supplementary material available at 10.1186/s12872-022-03005-w.

## Introduction

Heart Failure with preserved ejection fraction (HFpEF) is a highly prevalent yet under-diagnosed clinical syndrome [1, 2]. The hallmarks are the signs and symptoms of heart failure (HF) and a preserved left ventricular ejection fraction (LVEF). While the diagnosis of HFpEF is straightforward in acutely decompensated patients, stable euvolemic patients present a greater challenge [3]. Exertional dyspnea is non-specific and occurs in many other conditions. Specialist diagnostic tests e.g. expert echocardiography for diastolic dysfunction or invasive cardiac catheterization to document raised LV filling pressures may not be immediately available to the non-specialist. A recent study found that among more than 44,000 community-based patients likely to have HF, only 50% had a documented LVEF [4]. Furthermore, those eventually diagnosed as having HFpEF required many more pre-diagnosis investigations and consultations than HFrEF patients.

From a patient perspective, a diagnosis of HFpEF confers a high degree of morbidity as well as mortality rates equivalent to many forms of cancer [5]. Rates of readmission to hospital are high [6] and are associated with adverse outcomes [7]. From a healthcare system perspective, HFpEF is associated with significant costs due to frequent hospitalisation, with the median length of stay up to 19 days [8].

Until recently, no effective therapies were available for HFpEF [9–11], however recent clinical trial evidence suggests that sodium-glucose co-transporter 2 (SGLT-2) inhibitors are effective at decreasing hospitalization while increasing quality of life [12]. The presence of effective therapies highlights the need to identify patients who may derive benefit.

In previous epidemiological studies, identification and extraction of HFpEF cases from Electronic Health Records (EHR) has typically relied on diagnostic codes, additional medical record abstraction, and/or adjudication based on various expert criteria e.g. European Society of Cardiology criteria [13]. The EHR is however increasingly amenable to rapid and automated extraction of multiple clinical parameters, including the use of advanced natural language processing (NLP) algorithms to identify clinical concepts recorded in the unstructured text [14–16].

The aim of this study was to extract and analyze multi-modality data from the EHR using a machine learning approach to develop an automated prediction tool to identify patients likely to have HFpEF.

## Methods

### Derivation cohort

We performed a retrospective study using de-identified data of patients attending King’s College Hospital NHS Foundation Trust (KCH) in London (UK) between 2000 and 2019. We focused on patients who had undergone echocardiography as part of their inpatient or outpatient evaluation. With this starting point, a number of different patient cohorts were derived based on the LVEF, confirmed or possible HF, symptoms of dyspnea, and NT-proBNP (or BNP) level (see Additional file 1: Sections I and II). We identified confirmed HFpEF cases and two control cohorts: those with no evidence of HF (non-HF, Control cohort I) and those with HFrEF (Control cohort II). HFpEF cases were defined as patients with a preserved LVEF ≥ 50% (with no evidence of LVEF < 50% at any stage), a confirmed diagnosis of HF based on ICD10 codes I50.0, I50.1 or I50.9, dyspnea, and a raised NT-proBNP or BNP level (according to age-specific thresholds), in accordance with ESC diagnostic criteria [13]. Non-HF control cohort I was defined as no recorded diagnosis of HF, no dysponea, no reduced BNP and normal LVEF. HFrEF control cohort II was defined as having a recorded diagnosis of HF and reduced LVEF (i.e. < 50%). Patients with valvular heart disease (ICD10 codes I05-I09 and I35) were excluded.

### Test cohorts

We generated 4 test cohorts from patients who lacked at least one of the above diagnostic features for a confirmed diagnosis of HFpEF (see Additional file 1: Table S1 and Flowchart S1). We randomly sampled 100 patients from each of these four test subsets for analysis and removed samples where the clinical annotations disagreed or there was more than 70% missingness in signature predictors, leaving 269 in total.

### Data extraction and evaluation

Clinical and demographic data were retrieved from the structured and unstructured components of the EHR using the CogStack informatics platform [15]. Automated parsing of the EHR was achieved with a state-of-the-art enterprise search and well-validated natural language processing (NLP) tools, including MedCAT [16] and the Unified Medical Language System repository [17] as previously used by our group [18]. Clinical term extraction was restricted to concepts which represent clinical findings, diseases (apart from HF), medications, and signs and symptoms. This was linked to searches of structured data from an internal database containing echocardiographic data and ICD codes. Continuous variables were cleaned prior to cohort selection; e.g. conversion of text references of LVEF to numerical values and removal of measurement outliers (see Additional file 1: Section III). We used both platforms to arbitrate discrepancies in our derivation dataset as neither source proved to be comprehensive, in line with previous work [15, 16].

Echocardiographic data were based on formal studies performed according to British Society of Echocardiography guidelines (which are consistent with American and European guidelines) [19, 20]. In addition to collecting structured data from the echocardiographic dataset, we also collected numerical data that had been reporteded in the EHR text. For situations where a numerical value for LVEF had not been included in the echocardiogram report, we used a deep learning model to infer whether the LVEF was preserved based on written summary text of the echocardiogram report (see Additional file 1: Section III).

BNP or NT-proBNP results were obtained from samples drawn at any time in the study period and the maximum value for each subject was used.

All cases in the derivation dataset that were identified by the data pipeline as HFpEF were validated by manual review of the EHR by a cardiologist.

### Potential modeling predictors

A binary diagnostic outcome indicating the presence or absence of HFpEF was considered for modeling. Potential predictors to be included in a diagnostic signature included those used in previous HFpEF epidemiological studies [21, 22]. In addition, we adopted a comprehensive approach that included physiological variables, laboratory results, echocardiographic data and clinical concept references [23]. Structured data were collected within a two-month window around the last echocardiography result (or NTproBNP/BNP test result if available). Unstructured data were analyzed from the entire EHR prior to the date of the echocardiography result for each patient.

We made a second level predictor grouping according to whether the variables were initially recorded as (a) structured data: demographic and physiological parameters, and laboratory and echocardiography measurements; or (b) unstructured text in the EHR, extracted via the NLP platform. We adopted the bag-of-words [24] approach to transform clinical concept annotation into word vectors for modeling purposes. Concepts which were mentioned in < 10% of the derivation cohort were excluded. Data from the other predictor categories were collected and imputed prior to training, using the k-nearest neighbor (Scikit-learn python package v0.22) after min–max normalization. Following imputation, data items were rescaled into their original range to preserve the explainability of the final model.

### Data modeling, feature selection and validation

We used the tree-based multivariable extreme gradient boosting [25] algorithm (XGBoost, python package v0.9) for modeling, enabling inclusion of mixed data types and smooth handling of missing values and sparsity issues. As such, when a value is missing in the sparse predictor vector, the instance is classified into a default direction (see [25] for further details) that is learnt as optimal using derivation data.

SHAP [26] analysis (SHapley Additive exPlanations; SHAP python package v0.33) was used to order the predictors according to their prominence in discriminating cases from controls. Once the full model was created, we took a stepwise forward insertion scheme to include the more significant variables one at a time, in order to determine the minimal number of predictors that gave an acceptable performance relative to the use of all predictors. The final predictive models were trained and evaluated using the obtained optimal subset of predictors.

Model validation was undertaken in the test cohorts described earlier, using clinical assessment criteria from the H_2_FPEF score [3] as a comparator. A random sample of 400 patients from the test datasets was manually reviewed by two teams each comprising two cardiologists, in order to validate diagnoses. Any cases of clinician disagreement were removed from the evaluation, leaving a total of 269 patients in the test datasets (see Results, Table 1).

**Table 1:** Baseline characteristics of patients

	Non-HF controls (n = 194)	HFrEF controls (n = 1258)	HFpEF cases (n = 133)	P value cases versus controls	Test cohorts (n = 269, HFpEF cases = 68)			
					Set I (n = 61)	Set II (n = 68)	Set III (n = 71)	Set IV (n = 69)
Female, % (100%)	48.5%	36.8%	54.9%	–	61.8%	67.1%	68.9%	61.2%
Age, y (100%)	54 ± 18	69 (22)	73 ± 12	< 0.0001	66 ± 13	56 ± 15	55 ± 15	61 ± 13
Body mass index, kg/m^2^ (76%)	28.35 ± 8.07	28.75 ± 7.34	34.06 ± 10.07	< 0.0001	30.95 ± 8.15	32.18 ± 8.32	30.66 ± 7.58	31.67 ± 7.87
Hypertension, %	43.2%	81.6%	91.7%	–	83.8%	89.5%	67.6%	79.6%
Diabetes mellitus, %	20.1%	42%	54.1%	–	52.9%	31.6%	24.3%	34.7%
Atrial fibrillation, %	4.6%	47.6%	52.6%	–	50%	19.7%	6.7%	37.8%
Pulmonary hypertension, %	< 1%	12.2%	25.6%	–	26.5%	7.9%	2.7%	11.2%
Kidney disease, %	6.7%	35.5%	46.6%	–	66.1%	21.1%	24.3%	25.5%
Antihypertensive drugs, n**	–	–	–	–	2(10)	0 (4)	0(4)	0(0)
NT-proBNP, pg/ml (#)	46 (53)	138 (1676)	4181 (3620)	–	873 (1359)	282 (181)	NAN*	781 (1258)
BNP, pg/ml (#)	54 (73)	76 (353)	1510 (4488)	–	NAN*	NAN*	NAN*	796 (656)
Creatinine, umol/l (99%)	82.8 ± 39.7	88.0 (34.0)	84.0 (28.0)	0.165	89.0 (40.0)	78.0 (25.5)	78.5 (24.0)	86.6 ± 19.6
Hemoglobin, g/dl (96%)	12.6 ± 2.1	13.3 (2.6)	13.1 ± 1.8	0.836	12.7 ± 2.0	12.8 ± 1.7	12.6 ± 2.0	12.9 ± 2.1
White cell count, 10^9^/l (100%)	7.1 (4.33)	7.54 (3.99)	7.43 (3.76)	0.141	6.94 (3.57)	6.64 (3.4)	7.28 (4.43)	6.74 (3.16)
C-reactive protein, mg/l (96%)	6.5 ± 3.21	6.87 ± 3.12	7.4 (5.0)	0.254	6.93 ± 3.17	6.34 ± 3.01	6.62 ± 3.03	6.12 ± 3.11
Urea, mmol/l (99%)	5.73 ± 3.73	7.12 ± 4.43	6.4 (3.7)	0.687	6.85 (3.98)	5.3 (1.85)	4.65 (2.47)	5.95 (2.43)
Albumin, g/l (99%)	40.17 ± 6.98	41.13 ± 6.52	42.0 (3.0)	0.711	41.0 (6.0)	42.5 (4.25)	43.0 (6.0)	43.0 (3.0)
Sodium, mmol/l (99%)	138.34 ± 3.88	139.0 (4.0)	139.0 (3.0)	0.183	139.0 (3.25)	139.63 ± 2.52	139.34 ± 2.91	140.0 (3.0)
Potassium, mmol/l (99%)	4.57 ± 0.26	4.3 (0.6)	4.35 ± 0.58	0.720	4.2 (0.73)	4.28 ± 0.53	4.36 ± 0.52	4.31 ± 0.54
Calcium, mmol/l (98%)	2.28 (0.15)	2.29 (0.13)	2.31 ± 0.12	0.147	2.29 (0.17)	2.33 (0.14)	2.35 ± 0.13	2.34 ± 0.13
Systolic blood pressure, mmHg (63%)	132.93 ± 17.08	129.14 ± 21.9	139.54 ± 21.46	< 0.0001	140.89 ± 25.87	136.78 ± 17.84	138.63 ± 23.21	138.12 ± 18.65
Diastolic blood pressure, mmHg (67%)	79.15 ± 11.76	73.79 ± 13.36	74.96 ± 13.45	< 0.0001	73.16 ± 11.98	78.16 ± 13.09	80.68 ± 15.18	74.08 ± 13.73
Heart rate, beat/min (65%)	81.38 ± 14.28	73.69 ± 14.74	76.47 ± 16.72	0.0008	67.35 ± 9.63	75.31 ± 14.1	75.0 ± 8.8	74.64 ± 18.15
Oxygen saturation, % (52%)	98.1 ± 1.75	96.49 ± 5.05	96.22 ± 3.0	< 0.0001	96.36 ± 2.87	96.69 ± 2.87	97.89 ± 1.62	96.13 ± 4.66
LV end diastolic volume, ml (23%)	155.0 ± 61.89	152.46 ± 53.52	106.7 ± 26.52	< 0.0001	149.0 ± 16.97	124 ± NAN*	155.0 ± NAN *	110.83 ± 27.98
LV mass systolic, g (1%)	176.7 ± 53.0	265.2 ± 155.2	225.2 ± 74.5	< 0.0001	118.9 ± nan*	NAN *	210.3 ± 158.0	111.7 ± 89.9
LV ejection fraction, % (100%)	60.4 ± 3.9	44.2 ± 11.5	58.0 ± 4.9	< 0.0001	55.5 ± 2.1	60.5 ± 0.7	61.5 ± 2.1	55.3 ± 4.1
LV internal diameter at end diastole, cm/m^2^ (59%)	2.46 ± 0.24	2.71 ± 0.5	2.46 ± 0.36	0.0002	2.36 ± 0.26	2.46 ± 0.28	2.32 ± 0.24	2.45 ± 0.35
LV stroke volume, ml (4%)	92.5 ± 34.78	65.52 ± 19.78	55.0 ± 4.36	< 0.0001	82.0 ± 5.66	75.0 ± NAN *	93.0 ± NAN *	64.2 ± 19.7
LV outflow tract velocity time integral diameter, cm (20%)	2.13 ± 0.28	2.16 ± 0.24	2.17 ± 0.34	0.1126	2.03 ± 0.2	2.14 ± 0.24	2.13 ± 0.12	2.07 ± 0.23
LV end systolic volume, ml (22%)	62.5 ± 27.2	87.32 ± 42.81	42.67 ± 3.21	0.1708	66.5 ± 12.02	49.0 ± NAN *	62.0 ± NAN *	49.4 ± 10.45
LA systolic volume, ml (31%)	60.0 ± 19.52	86.47 ± 38.77	143.67 ± 70.44	< 0.0001	120.5 ± 28.99	112.0 ± NAN *	69.0 ± NAN *	70.33 ± 21.4
TR max PG, mmHg (80%)	26.7 ± 10.2	29.4 ± 11.2	34.16 ± 12.9	< 0.0001	37.2 ± 13.8	24.9 ± 10.18	26.3 ± 8.3	32.3 ± 11.4
E/e’ lateral ratio (50%)	7.26 ± 3.11	10.70 ± 6.07	11.59 ± 5.96	< 0.0001	13.54 ± 4.59	11.70 ± 5.16	9.35 ± 3.48	12.87 ± 7.27
E/e’ septal ratio (50%)	9.75 ± 4.99	14.51 ± 7.71	14.37 ± 5.6	< 0.0001	16.83 ± 5.44	14.77 ± 6.2	11.5 ± 4.52	16.04 ± 7.53
RV V1 max, cm/sec (6%)	82.28 ± 17.7	71.98 ± 22.65	80.41 ± 19.01	0.0001	95.37 ± 12.52	80.87 ± 17.42	71.34 ± 9.96	77.79 ± 18.88
RV V1 mean, cm/sec (4%)	47.04 ± 5.91	47.38 ± 14.19	52.03 ± 11.62	0.0048	53.5 ± 10.47	59.05 ± 5.18	48.65 ± 6.76	50.6 ± 9.53
Mitral valve E/A ratio, (84%)	1.08 ± 0.42	1.37 ± 0.07	1.13 ± 0.61	< 0.0001	1.23 ± 0.76	0.9 ± 0.34	0.98 ± 0.36	1.04 ± 0.4
Mitral regurgitation max velocity, cm/sec (10%)	483.46 ± 68.77	495.48 ± 88.56	502.84 ± 93.06	0.021	592.08 ± 98.05	553.39 ± 31.3	NAN	505.68 ± 119.98
Tricuspid regurgitation max velocity, cm/sec (80%)	233.23 ± 31.74	264.47 ± 56.62	274.1 ± 56.34	< 0.0001	310.38 ± 61.48	254.82 ± 53.76	239.73 ± 41.81	277.2 ± 50.81

The mean and SD (standard deviation) were obtained where the predictor distribution follows a normal distribution, whereas for predictors with a skewed distribution, the median and interquartile range (25th–75th) were used to report the statistics. To evaluate the distributional differences between cases and controls, the Mann–Whitney U test or the t test was acquired, where appropriate. Values in parentheses next to each predictor name indicate the data availability percentage

Set I: patients with normal EF, no/normal BNP record, a HF ICD10 code and at least one HF and dyspnea reference in their EHR

Set II: patients with normal EF, no/normal BNP record, no HF diagnostic code and at least one HF and dyspnea reference in their EHR

Set III: patients with normal EF, no BNP record, no HF diagnostic code nor HF reference in the EHR, at least one report of their dyspnea in their EHR

Set IV: patients with normal EF, raised BNP result with HF and dyspnea reference in their EHR but no HF diagnosis documented

(HF: heart failure, EF: ejection fraction, rEF: reduced EF, BNP: brain-natriuretic peptide test, EHR: electronic health record)

The following ICD10 codes were used to define the comorbidities:

Hypertension: I10-I15, I60-I69; Diabetes mellitus: E10-E14; Atrial fibrillation: I48; Pulmonary hypertension: I27; Kidney Disease: N18, N28, I12-I15

*Constraint-free assumption on our test sets resulted in predictors with either a singular value or a high proportion of missing values. In such cases, the computation of common statistics was not pragmatic and hence the NAN (Not A Number) value was reported, instead

**This predictor is only computed in the test cohort to enable the comparison with the H_2_FPEF score

#92.45% of HFpEF cases and controls had a BNP or pro-BNP level available

### Statistical analysis of predictors

Data are presented as mean and standard deviation (SD) or median and interquartile range (IQR) as appropriate. Differences between cases and controls were evaluated by the Mann–Whitney U test or unpaired t test, as appropriate. The area under the receiver-operating characteristic curve (AUROC), F1-score (macro and weighted) and average precision (AP) were used as performance metrics. The F1 score measures the performance of a classifer as the harmonic mean of precision (true positives as a proportion of all positive predictions) and recall (proportion of all positives correctly identified by the model), placing equal importance on both. Average precision is the weighted mean of precision scores obtained as the classification threshold is adjusted (therefore changing the model recall), with the change in recall used as the weight.

**Fig. 1:**
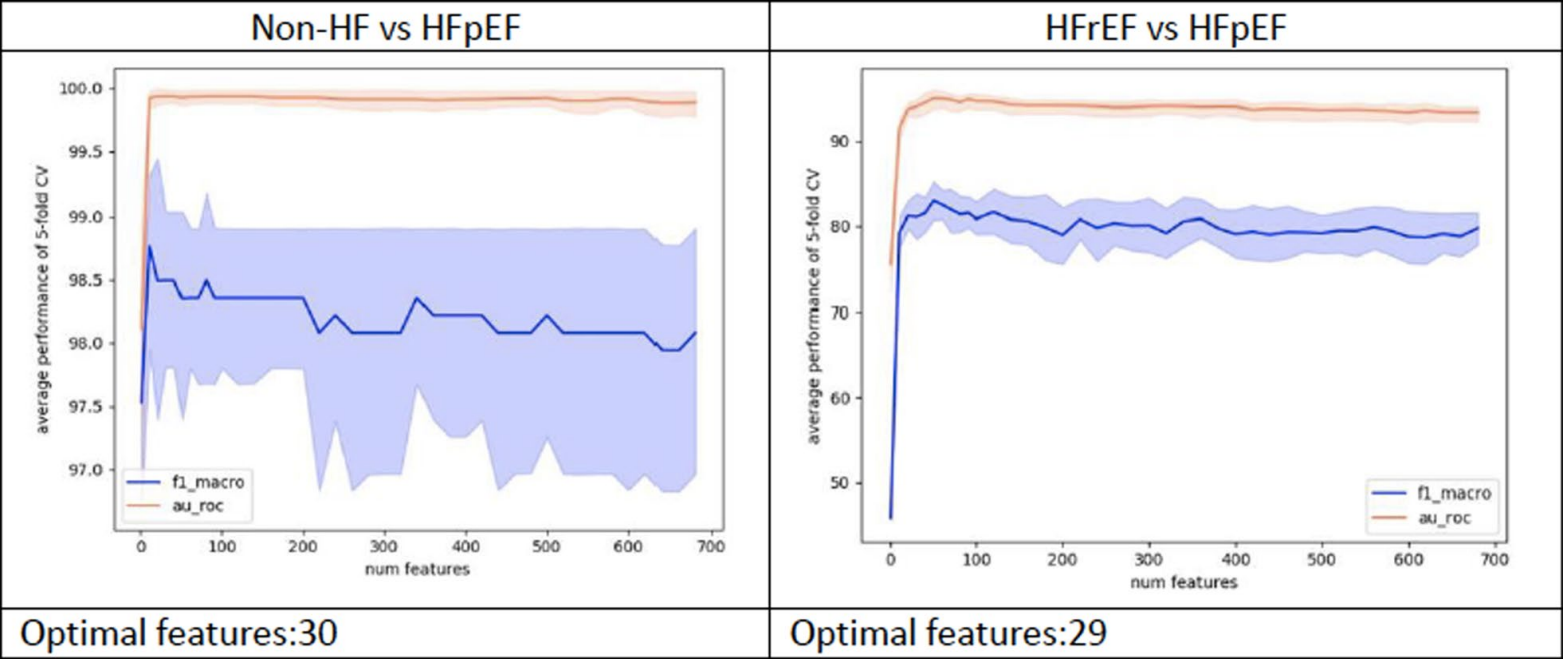
Feature selection analysis. Features were incrementally utilized for training the models to ensure a performance within ± 2 units of the AUROC and f1-macro metrics in fivefold cross-validation setup. Blue: f1-macro, Red: AUROC

A stratified fivefold cross-validation scheme (to ensure each fold is a good representative of the whole data in terms of class prevalence) was utilized for feature selection and derivation set validation. As such, the derivation data was divided into five subsets, four of which were used for training the model and the final one for validation/testing. The derivation and test subsets were shuffled until all five subsets were evaluated. The final performance was then reported as mean and standard deviation of all 5 tests (see Fig. 1).

The AUROC and AP were used as performance metrics and the Kappa statistic was used to measure the inter-rater agreement of proposed models. All tests were 2-sided, with P < 0.05 considered significant.

To evaluate the generalizability of the model to a new sample, Harrell optimism was calculated with 1000 boot-strap replicates [27]. To evaluate discrimination power of the proposed model beyond existing criteria, we compared the model’s AUROCs and AP performance against the recently proposed H_2_FPEF scoring system using the Random Forest (predecessor to XGBoost). We used a score of 3 points indicating > 50% probability of HFpEF.

Statistical analyses were performed in Python 3 using SciPy and Scikit-learn packages (v0.22).

## Results

1854 patients were included in the study of whom 1585 were in the derivation cohort (Table 1). HFpEF patients in the derivation cohort (n = 133) were older than those with HFrEF and those without heart failure (non-HF), with a higher proportion of females and a higher BMI. They also had a higher prevalence of hypertension, atrial fibrillation, diabetes and chronic kidney disease. Systolic and diastolic pressures were higher in the HFpEF group compared to HFrEF. Patients with HFpEF had lower end-diastolic and end-systolic volumes and higher septal E/e’ ratios than the non-HF control group.

### Diagnostic signatures for HFpEF diagnosis

Our first step was to determine model performance in predicting non-HF versus HFpEF and HFpEF versus HFrEF, and to identify the most useful features in each case.

**Fig. 2:**
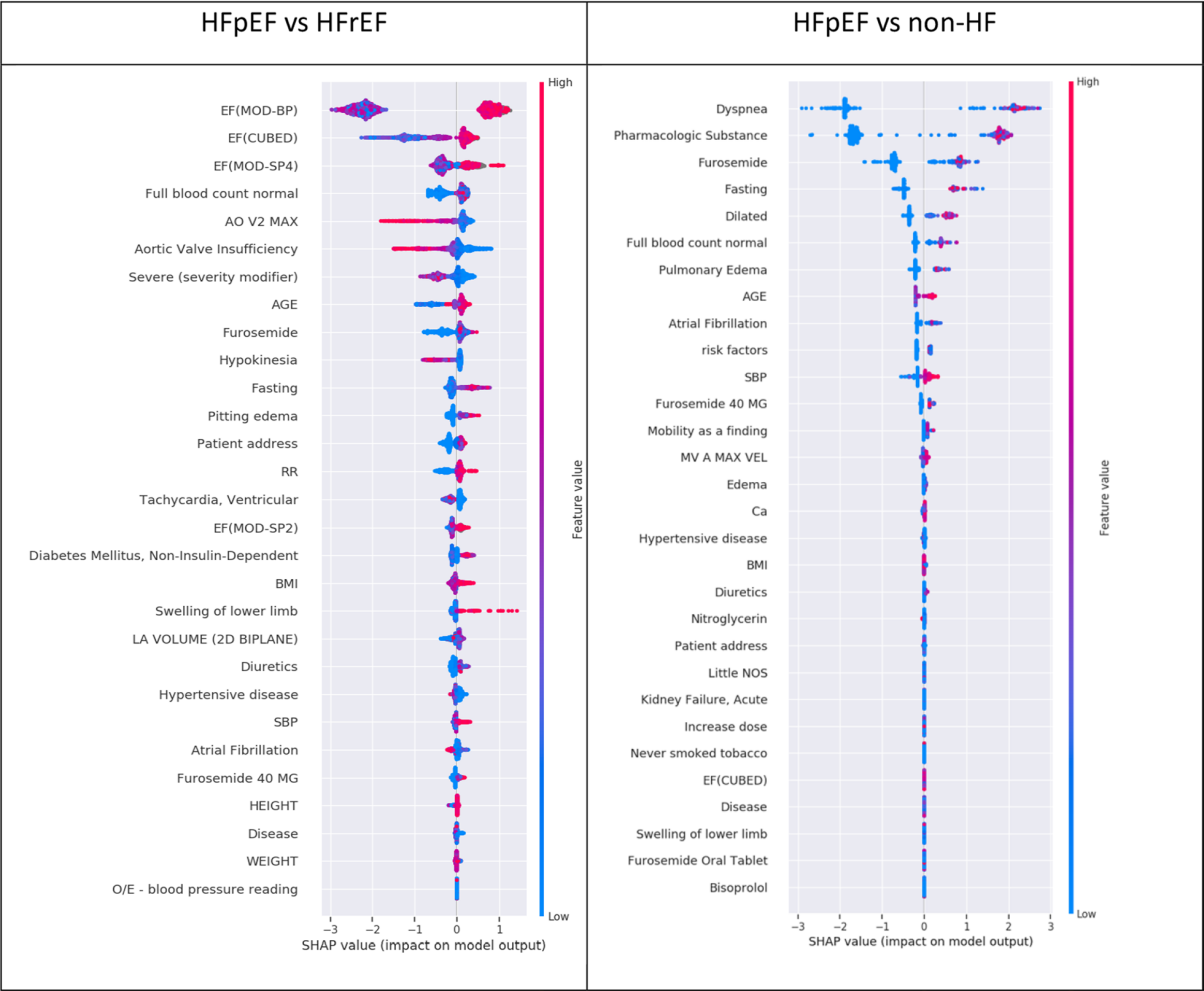
Feature importance using SHAP analysis in combined signatures. Denser distribution of red points at the positive quadrant of the plot is representative of higher values of a given predictor’s contribution in characterizing the positive class distribution i.e. in characterizing HFpEF. All feature names in upper case are structured features (blood tests, observations, echocardiogram values), all other features are derived from NLP

The minimum number of features required to distinguish HFpEF from non-HF was 30, while the minimum number required to distinguish HFpEF from HFrEF was 29. These features and their relative importance in discriminating HFpEF from non-HF and HFrEF are shown in Fig. 2. Dyspnea and ‘pharmacologic substance’ were the most prominent predictors in discrimination against non-HF whereas LVEF was most important for discrimination against HFrEF. However, many of the features (e.g. age, patient address) were common to the two groups. The text references to “patient address” and “pharmacologic substance” (detected when the text refers to medication) were interpreted as surrogate predictors of the number of complete hospital attendances. (Fig. 2).

We found that a combined model using both structured and unstructured data has better performance compared to using either structured or unstructured data alone (Table 2). This enhanced performance is more noted in discriminating HFpEF from HFrEF than discriminating HFpEF from non-HF (due to the dominancy of unstructured predictors in the HFpEF v non-HF model, see Fig. 2 and Table 3).

**Table 2:** Multivariable model performance using the fivefold cross-validation in derivation dataset

Model	Control set	f1_macro ± 95% CI	f1_weighted ± 95% CI	AUROC ± 95% CI
Structured signature	Non-HF	84.05 ± 2.7	84.18 ± 2.7	92.04 ± 1.4
	HFrEF	75.75 ± 2.1	87.22 ± 1.42	90.31 ± 3.5
Unstructured signature	Non-HF	98.81 ± 1.3	98.82 ± 1.3	99.7 ± 0.5
	HFrEF	78.59 ± 4.9	88.99 ± 2.1	94.38 ± 1.4
Combined signature	Non-HF	98.57 ± 1.4	98.59 ± 1.4	99.8 ± 0.3
	HFrEF	83.03 ± 2.8	90.91 ± 1.6	95.67 ± 2.0

**Table 3:** Additive SHAP feature importance for each category of predictors in the combined signatures

	Model	Unstructured data	Structured data			
		Symptoms	Echocardiography parameters	Vitals	Age & Sex	Lab results
Summed importance of grouped features	HFpEF versus non-HF	0.953	0.036	0.011	0.033	< 0.001
	HFpEF versus HFrEF	0.551	0.334	0.115	0.058	< 0.001

### Selection of the final model and evaluation in test cohorts

The final model that was used for test evaluations aggregates the HFpEF versus HFrEF and HFpEF versus non-HF signature likelihood predictions through an averaging operation. It therefore uses all features from both component models (Table 4). In the final “aggregated” model, a patient is predicted to have HFpEF if the average predicted probability of HFpEF versus non-HF and versus HFrEF is >  = 0.5. The idea of the aggregated model is to aid discrimination between HFpEF and related conditions. We used this aggregate model to make predictions on the test sets. Additional file 1: Figure S5 summarises the entire processing and model training pipeline, while Additional file 1: Figure S6 gives details of model adaptation [28].

**Table 4:** All variables used in the final model

Variable	HFpEF versus HFrEF	HFpEF versus non-HF
AGE	1	1
AO V2 MAX	1*	0
Aortic Valve Insufficiency	1	0
Atrial Fibrillation	1	1
BMI	1	1
Bisoprolol	0	1
Ca	0	1
Diabetes Mellitus, Non-Insulin-Dependent	1	0
Dilated	0	1*
Disease	1	1
Diuretics	1	1
Dyspnea	0	1*
EF(CUBED)	1*	1
EF(MOD-BP)	1*	0
EF(MOD-SP2)	1	0
EF(MOD-SP4)	1*	0
Edema	0	1
Fasting	1	1*
Full blood count normal	1*	1
Furosemide	1	1*
Furosemide 40 MG	1	1
Furosemide Oral Tablet	0	1
HEIGHT	1	0
Hypertensive disease	1	1
Hypokinesia	1	0
Increase dose	0	1
Kidney Failure, Acute	0	1
LA VOLUME (2D BIPLANE)	1	0
Little LOS	0	1
MV A MAX VEL	0	1
Mobility as a finding	0	1
Never smoked tobacco	0	1
Nitroglycerin	0	1
O/E—blood pressure reading	1	0
Patient address	1	1
Pharmacologic Substance	0	1*
Pitting edema	1	0
Pulmonary Edema	0	1
RR	1	0
SBP	1	1
Severe (severity modifier)	1	0
Swelling of lower limb	1	1
Tachycardia, Ventricular	1	0
WEIGHT	1	0
Risk factors	0	1

The columns “HFpEF versus HFrEF” and “HFpEF versus non-HF” indicate use of a variable in either model where 1 = used and 0 = unused. Asterisks indicate the top 5 most important variables in each model according to SHAP analysis. All variables in upper case are structured features, all other features are derived from NLP

**Fig. 3:**
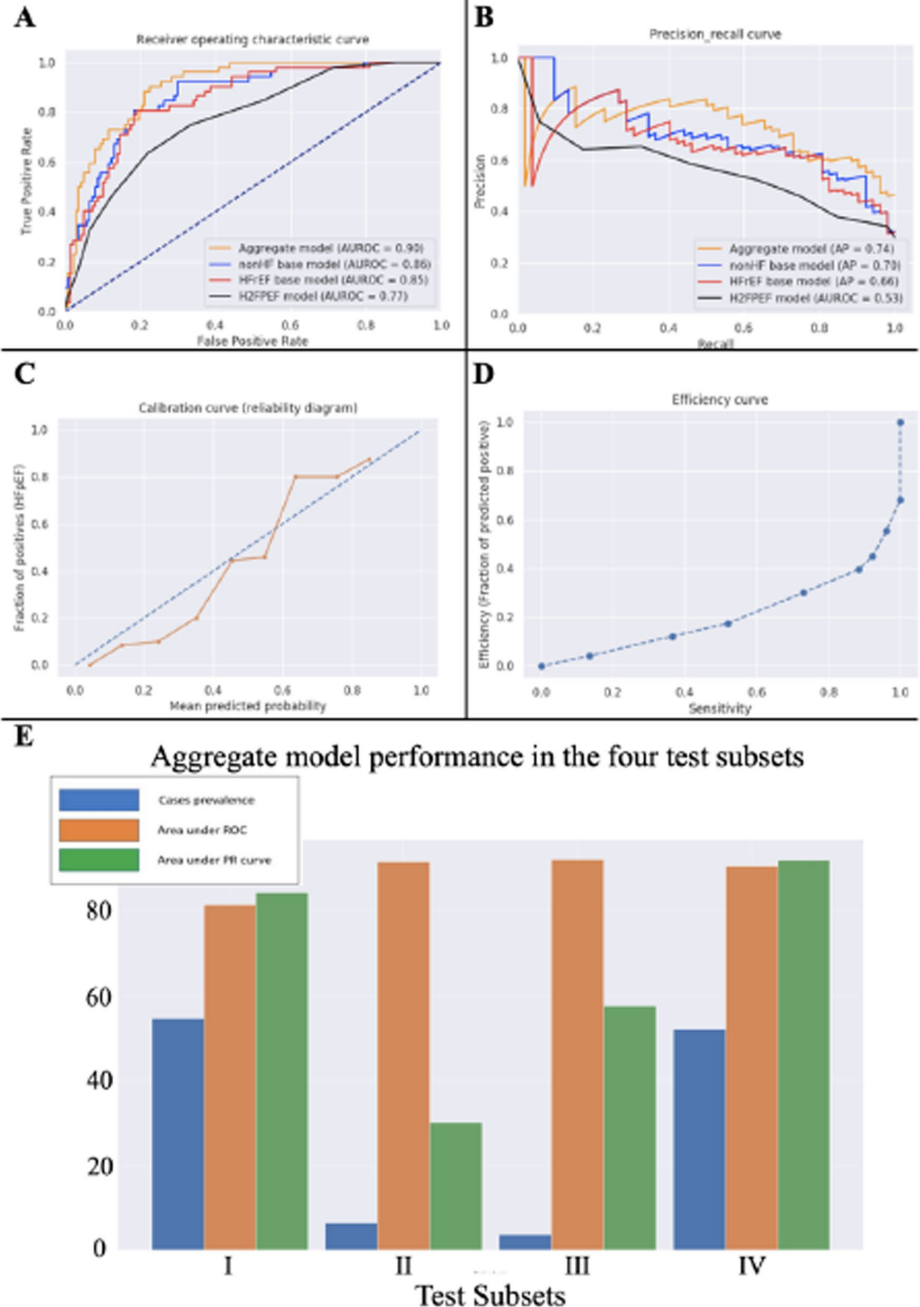
Performance of base and aggregate models. Panel **A**: Receiver Operating Characteristic curves for base models, aggregate model, and H_2_FPEF score. Panel **B**: Precision Recall curves for base models, aggregate model, and H_2_FPEF score. Panel **C**: Calibration curve for aggregate model. Panel **D**: Efficiency curve for aggregate model. Panel **E**: Aggregate model performance in the 4 test subsets

The performance of both proposed base models and the final aggregated model remained robust in the test cohort as compared to expert clinical consensus, with an AUROC performance of 0.86 (95% CI  ± 0.002) and 0.85 (95% CI  ± 0.001) in HFpEF vs non-HF and HFpEF versus HFrEF models, respectively and an enhanced aggregate performance of 0.90 (95% CI  ± 0.002) in our final aggregate model (Fig. 3).

Lastly, we compared the final aggregate model as well as the baseline HFpEF versus non-HF and HFpEF versus HFrEF models with the recently described H_2_FPEF model. The AUROC and average precision of both the aggregate model and the individual baseline models was higher than the H_2_FPEF model (Table 5). We additionally used the Cohen’s kappa score to report on the agreement between the predictions made by our proposed baseline HFpEF versus non-HF and HFpEF versus HFrEF models to better highlight the efficiency of the aggregate model over the individual base models discriminating HFpEF from non-HF and HFrEF. The positive kappa score of 0.3 indicates a weak agreement between the two base models (i.e. can make different predictions for whether HFpEF is present in the same patient). This was expected as the test cohort had lower availability of clinical assessments compared to the derivation cohort. Together with the improved overall performance, this result supports the use of the aggregated model.

**Table 5:** Multivariable model performance in independent test cohort

	Performance metric	H_2_FPEF, %	Combined HFpEF versus non-HF signature, %	Combined HFpEF versus HFrEF signature, %	Aggregate model HFpEF versus HFrEF/non-HF score, %	Scoring Agreement* (HFpEF versus HFrEF and HFpEF versus non-HF)
Test set	AUROC (95% CI)	0.77	0.86 (± 0.002)	0.85 (± 0.001)	0.90 (± 0.002)	0.3
	AP	0.53	0.70	0.66	0.74	

The 95% CI is reported using bootstrapping in a thousand of iterations

*AUROC* area under receiver operative curve; *AP* average precision; *CI* confidence interval in bootstrapped samples

*HFpEF annotation agreement between the two scoring systems using Cohen’s kappa statistics (python 3, Sklearn v.0.22)

## Discussion

In this study, we have developed an automated pipeline for EHR-based data collection, processing and modeling to identify patients with a high likelihood of HFpEF. We incorporated multi-modality data, including both structured and unstructured predictors, to generate a disease diagnostic signature. The proposed signature was validated in a separate cohort of patients and performed favourably as compared either to expert clinical consensus or the recently proposed H_2_FPEF score [3].

Analysis of the signatures that distinguished HFpEF from non-cardiac causes of dyspnea (non-HF) revealed anticipated predictors such as atrial fibrillation, hypertension, diabetes mellitus, kidney failure and obesity, in accordance with previous literature [3]. In addition, surrogate measures of multiple previous clinical encounters detected by the NLP algorithm as frequent text references to terms such as “pharmacologic substance” (a reference to drug treatment but not a specific medication) or “patient address” were very useful. This may reflect the fact that patients with HFpEF may require multiple clinical visits and investigations, often with different specialities, before a diagnosis is established [4]. Apart from LVEF itself, features that distinguished HFpEF from HFrEF included age, peripheral edema, and other echocardiographic measures. An advantage of the approach that we employed may be that it is unbiased and comprehensive and identifies variables for inclusion in the diagnostic signature based purely on the results of the objective feature selection process. This may be one reason why our algorithm outperforms the H_2_FPEF score, which is based on the evaluation of selected variables rather than a comprehensive unbiased analysis. In this regard, it is of interest that echocardiographic predictors that contributed to the differentiation of HFpEF from HFrEF included maximum flow velocity across the aortic valve, aortic insufficiency and LA volume whereas E/e’ (which is part of the H_2_FPEF score) did not feature in the selected predictors. Indeed, we note that several indices from a standard echocardiographic dataset that are typically used to identify HFpEF do not feature as predictors differentiating HFpEF from HFrEF. These include LV cavity dimensions; LV wall thickness and mass; and E/e’ as mentioned above. However, given the defining features of HFpEF versus HFrEF, it is perhaps not surprising that the top differentiating features are variations of quantifying LVEF.

A major underlying problem in efforts to develop or test new treatments for HFpEF is the difficulty in consistently diagnosing the syndrome [4]. Many different approaches are used in the literature based on varying criteria published by national and international societies, and diverse inclusion criteria have been used in clinical trials [29–31]. The problem is compounded by the likelihood that HFpEF is a heterogenous syndrome in which sub-populations may have differing underlying pathophysiology and outcomes [21, 22, 29]. The approach we present enables rapid identification of likely HFpEF cases among which further specific phenotyping could be performed to refine the diagnosis and potentially test or target defined interventions, or to identify potential subjects for research studies. In practice, the output of each of our models is a predicted probability in the range 0–1, for example the HFpEF vs non-HF model could return 0.89, indicating a predicted 89% probability of HFpEF. Importantly, this approach aims to identify both compensated and decompensated HFpEF cases, using an automated and data-driven approach that is effective even where structured data (e.g. NT-proBNP measurements) are scarce. The approach may be considered complementary to scores such as H_2_FPEF. Our signature is ideally suited to rapidly identify a large number of possible HFpEF cases from EHR whereas H_2_FPEF is better suited for use by the clinician evaluating an individual patient who is suspected to have HFpEF.

This study is the first to use SHAP analysis for feature selection in this context. We comprehensively validated all variations of the derived models in multiple datasets with underlying variational distributions. We demonstrated a significant improvement in HFpEF diagnostic performance when discriminating the patients with HFpEF from those with HFrEF or no HF history. A key strength of our approach is that modeling numerical assessment data (structured results signature) and EHR concept references separately makes the models applicable in scenarios where one of these sources of data may be scarce. Moreover, the dual modeling of HFpEF separation from non-HF and HFrEF subjects increases the utility of the proposed pipeline in distinguishing among a wider group of clinical conditions.

### Limitations

The UMLS clinical concept encoding that was used to extract unstructured observations does not support distinct encoding of different disease stages and could therefore cause some inaccuracy. In a more general aspect, the a priori assumptions that we made to identify definite HFpEF cases in the derivation dataset influenced the characterisation of the cohort. For example, we utilised ICD-10 diagnostic codes in the identification of patients with heart failure. Previous studies have demonstrated inaccuracy in identifying incident heart failure using ICD-10 coding as the sole source [32]. It is possible that such inaccuracy is present in our coding system; however the use of additional features (symptoms, LVEF, BNP/NTproBNP) in case classification mitigates this risk in our study. Similarly, it is possible that for some patients an HF diagnosis is known but not recorded in the records we accessed, or was recorded but not detected by our NLP algorithm (i.e. a false negative). As we combined a number of other features, including symptoms and blood tests, in assigning our final HF diagnosis labels, we expect the overall impact on the results to be minimal.

The inclusion of a raised BNP criterion restricts the cohort to a subgroup of HFpEF subjects (a proportion of HFpEF patients have a normal BNP), which was evident in test cohorts where many of the subjects did not have BNP measurements. This issue could be successfully handled through transfer learning techniques but would require some labelled data from a new domain to facilitate such a feedback training loop. The choice of data imputation technique could be another source of minor but systematic error. The discriminant power of the model to detect HFpEF is lower in test subsets where the missing data rate is higher and HFpEF cases are a small proportion of the overall number. Finally, the applicability of our model in patients with HFpEF who have never required hospital evaluation or admission is unknown. However, a strength of our approach is that a dedicated specialist assessment for HF is not required to assess the probability of HFpEF among patients undergoing general hospital evaluation (e.g. non-cardiological), even in the absence of commonly used diagnostic data such as NTproBNP levels. The lack of independent validation is a limitation of this study. Evaluation of the derived model’s performance in independent datasets from other centres and in community-based datasets will be informative in future studies. Although we compared performance of the model with the H_2_FPEF score [3], due to its stated aim of estimating the likelihood that HFpEF among patients with unexplained dyspnea to guide further testing, we did not compare performance to the HFA-PEFF algorithm which is a multi-step diagnostic algorithm [33]. Furthermore, the comparison of our algorithm’s performance with the H_2_FPEF should be confirmed in a separate validation cohort.

The HFrEF group (Control Cohort II) comprised patients with a diagnosis of HF and reduced LVEF on echocardiogram using a cut-off value of < 50%. As such, this cohort combines HFrEF and HFmrEF cases as decribed in ESC guidelines [13]. Finally, in our analysis we focus on performance at the group level. Future work should establish the applicability of this method on an individual level, such as focusing on older or younger patients.

## Conclusion

In this study, we have developed a rapid and automated data-driven approach that is effective at identifying patients from EHR who are likely to have HFpEF. This algorithm affords significant potential to rapidly identify patients for more detailed analyses and access to evidence-based therapies that are known to improve quality of life and decrease rates of hospitalisation. The approach that we report could in principle be readily applied to other diseases and conditions that are similarly difficult to diagnose.

## Supplementary Information

**Additional file 1**. Supplementary Methods.

## Data Availability

The datasets analysed during the current study are not publicly available due to hospital information governance regulations but are available from the corresponding author on reasonable request. We will share our models and the analytical methods to facilitate the replication of the study on data collected from other hospitals.

## References

[1] Owan TE, Hodge DO, Herges RM, Jacobsen SJ, Roger VL, Redfield MM (2006). Trends in prevalence and outcome of heart failure with preserved ejection fraction. N Engl J Med.

[2] Bursi F, Weston SA, Redfield MM, Jacobsen SJ, Pakhomov S, Nkomo VT, Meverden RA, Roger VL (2006). Systolic and diastolic heart failure in the community. JAMA.

[3] Reddy YNV, Carter RE, Obokata M, Redfield MM, Borlaug BA (2018). A simple, evidence-based approach to help guide diagnosis of heart failure with preserved ejection fraction. Circulation.

[4] Huusko J, Purmonen T, Toppila I, Lassenius M, Ukkonen H (2020). Real-world clinical diagnostics of heart failure patients with reduced or preserved ejection fraction. ESC Heart Fail.

[5] Dunlay SM, Roger VL, Redfield MM (2017). Epidemiology of heart failure with preserved ejection fraction. Nat Rev Cardiol.

[6] Shah KS, Xu H, Matsouaka RA, Bhatt DL, Heidenreich PA, Hernandez AF, Devore AD, Yancy CW, Fonarow GC (2017). Heart failure with preserved, borderline, and reduced ejection fraction: 5-year outcomes. J Am Coll Cardiol.

[7] Huusko J, Tuominen S, Studer R, Corda S, Proudfoot C, Lassenius M, Ukkonen H (2020). Recurrent hospitalizations are associated with increased mortality across the ejection fraction range in heart failure. ESC Heart Fail.

[8] Shiga T, Suzuki A, Haruta S, Mori F, Ota Y, Yagi M, Oka T, Tanaka H, Murasaki S, Yamauchi T (2019). Clinical characteristics of hospitalized heart failure patients with preserved, mid-range, and reduced ejection fractions in Japan. ESC Heart Fail.

[9] Yusuf S, Pfeffer MA, Swedberg K, Granger CB, Held P, McMurray JJ, Michelson EL, Olofsson B, Ostergren J, Investigators C (2003). Effects of candesartan in patients with chronic heart failure and preserved left-ventricular ejection fraction: the CHARM-preserved trial. Lancet.

[10] Solomon SD, McMurray JJV, Anand IS, Ge J, Lam CSP, Maggioni AP, Martinez F, Packer M, Pfeffer MA, Pieske B (2019). Angiotensin–neprilysin inhibition in heart failure with preserved ejection fraction. N Engl J Med.

[11] Pitt B, Pfeffer MA, Assmann SF, Boineau R, Anand IS, Claggett B, Clausell N, Desai AS, Diaz R, Fleg JL (2014). Spironolactone for heart failure with preserved ejection fraction. N Engl J Med.

[12] Anker SD, Butler J, Filippatos G, Ferreira JP, Bocchi E, Bohm M, Brunner-La Rocca HP, Choi DJ, Chopra V, Chuquiure-Valenzuela E (2021). Empagliflozin in heart failure with a preserved ejection fraction. N Engl J Med.

[13] Ponikowski P, Voors AA, Anker SD, Bueno H, Cleland JG, Coats AJ, Falk V, Gonzalez-Juanatey JR, Harjola VP, Jankowska EA (2016). 2016 ESC guidelines for the diagnosis and treatment of acute and chronic heart failure: the task force for the diagnosis and treatment of acute and chronic heart failure of the European Society of Cardiology (ESC) developed with the special contribution of the heart failure association (HFA) of the ESC. Eur Heart J.

[14] Wu H, Toti G, Morley KI, Ibrahim ZM, Folarin A, Jackson R, Kartoglu I, Agrawal A, Stringer C, Gale D (2018). SemEHR: a general-purpose semantic search system to surface semantic data from clinical notes for tailored care, trial recruitment, and clinical research. J Am Med Inform Assoc.

[15] Jackson R, Kartoglu I, Stringer C, Gorrell G, Roberts A, Song X, Wu H, Agrawal A, Lui K, Groza T (2018). CogStack-experiences of deploying integrated information retrieval and extraction services in a large national health service foundation trust hospital. BMC Med Inform Decis Mak.

[16] Kraljevic ZST, Shek A, Roguski L, Noor K, Bean D, Mascio A, Zhu L, Folarin AA, Roberts A, Bendayan R, Richardson MP, Stewart R, Shah AD, Wong WK, Ibrahim Z, Teo JT, Dobson RJB (2021). Multi-domain clinical natural language processing with MedCAT: the medical concept annotation toolkit. Artif Intell Med.

[17] (MD) B. UMLS reference manual. 2009.

[18] Bean DM, Teo J, Wu H, Oliveira R, Patel R, Bendayan R, Shah AM, Dobson RJB, Scott PA (2019). Semantic computational analysis of anticoagulation use in atrial fibrillation from real world data. PLoS ONE.

[19] Wharton G, Steeds R, Allen J, Phillips H, Jones R, Kanagala P, Lloyd G, Masani N, Mathew T, Oxborough D (2015). A minimum dataset for a standard adult transthoracic echocardiogram: a guideline protocol from the British Society of Echocardiography. Echo Res Pract.

[20] Lang RM, Badano LP, Mor-Avi V, Afilalo J, Armstrong A, Ernande L, Flachskampf FA, Foster E, Goldstein SA, Kuznetsova T (2015). Recommendations for cardiac chamber quantification by echocardiography in adults: an update from the American Society of Echocardiography and the European Association of Cardiovascular Imaging. Eur Heart J Cardiovasc Imaging.

[21] Shah SJ, Katz DH, Selvaraj S, Burke MA, Yancy CW, Gheorghiade M, Bonow RO, Huang CC, Deo RC (2015). Phenomapping for novel classification of heart failure with preserved ejection fraction. Circulation.

[22] Shah SJ, Kitzman DW, Borlaug BA, van Heerebeek L, Zile MR, Kass DA, Paulus WJ (2016). Phenotype-specific treatment of heart failure with preserved ejection fraction: a multiorgan roadmap. Circulation.

[23] Bielinski SJ, Pathak J, Carrell DS, Takahashi PY, Olson JE, Larson NB, Liu H, Sohn S, Wells QS, Denny JC (2015). A robust e-epidemiology tool in phenotyping heart failure with differentiation for preserved and reduced ejection fraction: the electronic medical records and genomics (eMERGE) network. J Cardiovasc Transl Res.

[24] Major V, Surkis A, Aphinyanaphongs Y. Utility of general and specific word embeddings for classifying translational stages of research. In: AMIA Annual Symposium Proceedings. 2018**;2018**:1405–1414. PMC6371342 30815185

[25] Chen T. GC: XGBoost: a scalable tree boosting system. KDD ’16: Proceedings of the 22nd ACM SIGKDD international conference on knowledge discovery and data mining. 2016:785–794.

[26] Lundberg S. S-IL: A unified approach to interpreting model predictions. NIPS. 2017.

[27] Steyerberg EW, Harrell FE, Borsboom GJ, Eijkemans MJ, Vergouwe Y, Habbema JD (2001). Internal validation of predictive models: efficiency of some procedures for logistic regression analysis. J Clin Epidemiol.

[28] Donahue J, Hoffman J, Rodner E, Saenko K, Darrell T. Semi-supervised domain adaptation with instance constraints. In: 2013 IEEE conference on computer vision and pattern recognition. 3012:668-675.

[29] Pfeffer MA, Shah AM, Borlaug BA (2019). Heart failure with preserved ejection fraction in perspective. Circ Res.

[30] Parikh KS, Sharma K, Fiuzat M, Surks HK, George JT, Honarpour N, Depre C, Desvigne-Nickens P, Nkulikiyinka R, Lewis GD (2018). Heart failure with preserved ejection fraction expert panel report: current controversies and implications for clinical trials. JACC Heart Fail.

[31] Ho JE, Zern EK, Wooster L, Bailey CS, Cunningham T, Eisman AS, Hardin KM, Zampierollo GA, Jarolim P, Pappagianopoulos PP (2019). Differential clinical profiles, exercise responses, and outcomes associated with existing HFpEF definitions. Circulation.

[32] Kaspar M, Fette G, Guder G, Seidlmayer L, Ertl M, Dietrich G, Greger H, Puppe F, Stork S (2018). Underestimated prevalence of heart failure in hospital inpatients: a comparison of ICD codes and discharge letter information. Clin Res Cardiol.

[33] Pieske B, Tschope C, de Boer RA, Fraser AG, Anker SD, Donal E, Edelmann F, Fu M, Guazzi M, Lam CSP (2020). How to diagnose heart failure with preserved ejection fraction: the HFA–PEFF diagnostic algorithm: a consensus recommendation from the Heart Failure Association (HFA) of the European Society of Cardiology (ESC). Eur J Heart Fail.

