# Supplementary material for: Diagnostic signature for heart failure with preserved ejection fraction (HFpEF): a machine learning approach using multi-modality electronic health record data
Source: BMC Cardiovasc Disord. 2022 Dec 26;22:567. doi: 10.1186/s12872-022-03005-w (PMC9791783; doi:10.1186/s12872-022-03005-w)
Supplement: Supplementary file 1 — Additional file 1. Supplementary Methods. [file 12872_2022_3005_MOESM1_ESM.pdf]

Sec. I Diagnosis assertion criteria

Table S1. *Data availability table used for case and control definition and cohort selection. Black shaded cells indicate the condition is true, white cells indicate the condition is false. Grey shade indicates partial or unknown validity e.g. n/rBNP for test set I and II where no BNP result was available so we do not know if the BNP is normal or reduced. HF: heart failure, Diag: ICD10 diagnosis, cDiag: complementary diagnosis derived from semi-structured data through NLP, pEF: preserved EF (>=50), rEF: reduced EF (<50), nBNP: normal BNP, rBNP: abnormal BNP*

Sets	HF concept ref.	Diag	cDiag	pEF	rEF	nBNP	rBNP	Dysp
**DERIVATION COHORT**								
Confirmed HFpEF								
Control I (Non-HF)								
Control II (HFrEF)								
**TEST COHORT**								
Test subset I								
Test subset II								
Test subset III								
Test subset IV								

Flowchart S1. *The cohort definition flowchart*

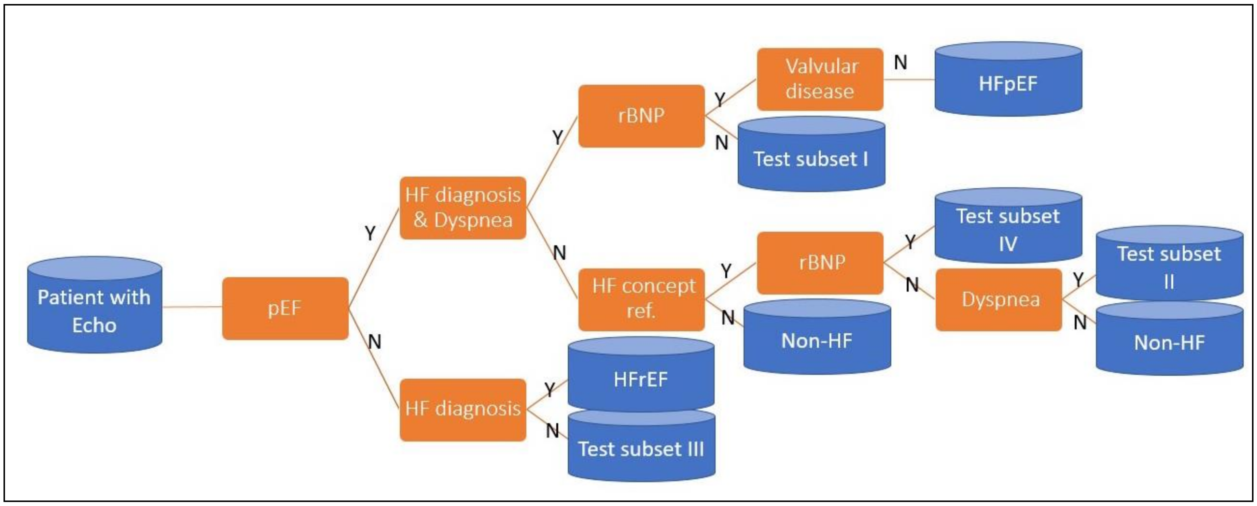

Figure S1. *Age and gender histogram in the HFpEF cases of derivation set.*

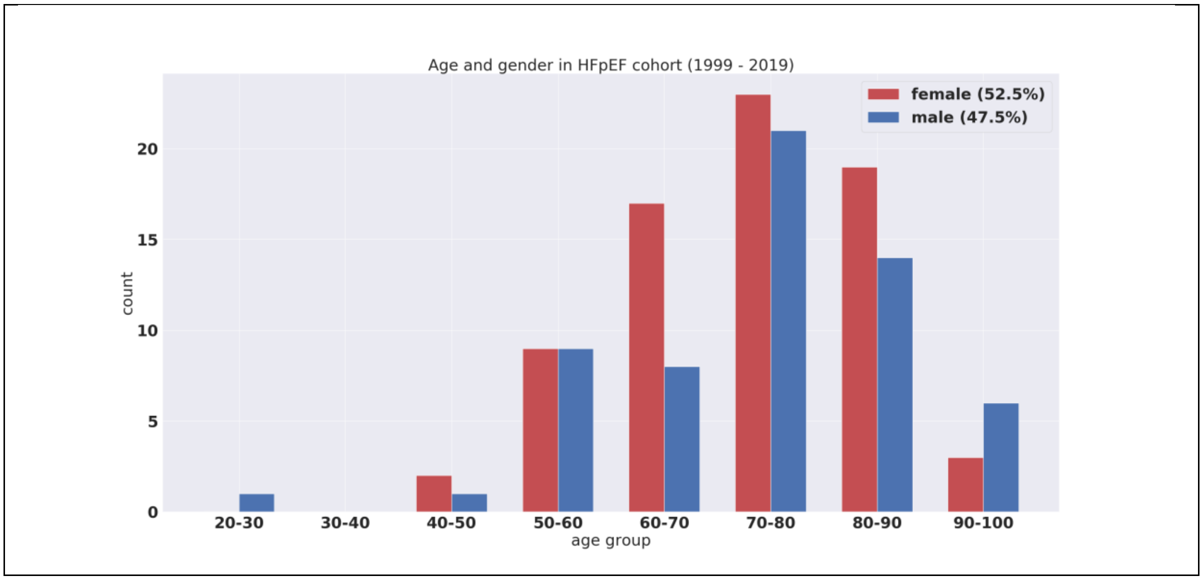

Sec. II: BNP/NT-proBNP assessment criteria

Alg.1. *Algorithm which was used to define the BNP and NT-pro-BNP values as normal or abnormal for the study*

```
Inputs: TEST_TYPE, BNP_VALUE, PATIENT_AGE
Output: BNP_state

If TEST_TYPE is NT-proBNP:
        If (BNP_VALUE >= 125) & (PATIENT_AGE <75):
                BNP_state <-- abnormal
        Else if (BNP_VALUE>=450) & (PATIENT_AGE >=75):
                BNP_state <-- abnormal
        Else:
                BNP_state <-- normal
If TEST_TYPE is BNP:
        If (BNP_VALUE >= 100) & (PATIENT_AGE <75):
                BNP_state <-- abnormal
        Else if (BNP_VALUE>=130) & (PATIENT_AGE >=75):
                BNP_state <-- abnormal
        Else:
                BNP_state <-- normal
Return BNP_state
```

Sec. III: Ejection fraction aggregation across structured and unstructured data

In order to extract all EF values in patient's historical records, we used two sources of data; 1) structured data containing echocardiographic measurements of in-hospital Ultrasounds scans and 2) unstructured free-text reports of the ultrasound results obtained in other hospitals and included in patient's EPR.

Extracting the EF values from structured data is done through SQL queries. The EF info in the text, however encompassed in three main formats, i) numeric EF reported within the text, ii) semi-structured fields inserted as text in the EPR document and iii) the clinical interpretation on normality of the EF value is reported in free-text (Figure S2 depicts these variation).

To cope with all variations, we used three parallel streams of natural language processing units. The first processing units (PU) extracts the numeric reports of the EF measurements in a word-window around the EF reference in the text using NLTK python toolbox. The second unit uses a rule-based approach to re-structure the semi-structured echocardiographic reports into a SQL table. The numerical results of the first two units are aggregated with the structured EF values on subject level.

For subjects who had no numerical EF values extracted but had echocardiographic interpretation summary, we used a three-layers LSTM network to infer the EF deviation from normality (positive class: EF>=50%, negative class: EF<50%).

We have tested the performance of the processing units on the mutually available reports in both structured and unstructured data sources.

The performance of our proposed deep learning approach is compared (Figure S3) with a decision tree model using the three-fold cross validation of ROC curves and on 2000 manually annotated samples. Finally, the staged aggregation evaluations are reported in Figure S4 using the subset of subjects who had EF assessment data availability across the two structured (Xcelera, SQL) and unstructured (EPR free-text, ELK) databases.

Figure S2. *Various formats in which the EF value is reported in unstructured EPR reports. EF: Ejection fraction*

Interpretation Summary

A two-dimensional transthoracic echocardiogram with M-mode and Doppler was performed.
Normal biventricular size and systolic function. Other chambers and all valves are structurally normal.
Right ventricular systolic pressure is estimated at 40-45mmHg.

Left Ventricle
The left ventricle is normal in size. There is normal left ventricular wall thickness. Left ventricular systolic function is normal. Estimated LVEF ~64% by Simpson's bi-plane method.
Abnormal Diastolic Function Grade I - Impaired Relaxation with normal filling pressures.
The left ventricular wall motion is normal....

Measurments

LVIDd: 3.9 cm (3.9-5.3 cm)

LVIDs: 2.5 cm (2.5-4.5 cm)

FS: 35.4 % (27-45%)

IVSd: 0.92 cm (0.8-1.1 cm)

LVPWd: 0.71 cm (0.5-1.1 cm)

LA dimension: 3.0 cm (2.7-3.8 cm)

DV(MOD-sp2): 105.9 ml

EDV(sp2-el): 102.4 ml

LVLs ap2: 6.6 cm

EF(MOD-sp2): 66.2 %

EF(sp2-el): 66.5 %

EF(MOD-bp): 64.3 %
...

Figure S3. *Deep learning model performance on discriminating the text reference of a preserved/normal ejection fraction from the reduced fraction.*

Transductive LSTM

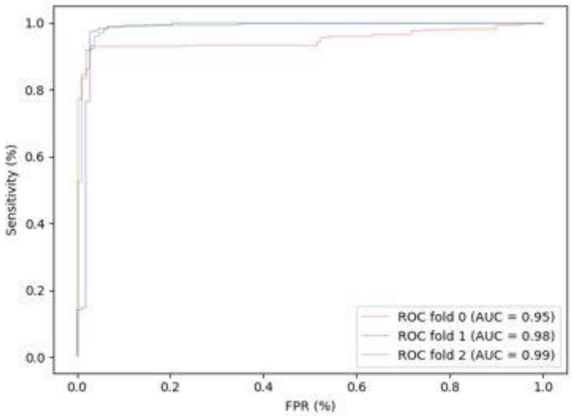

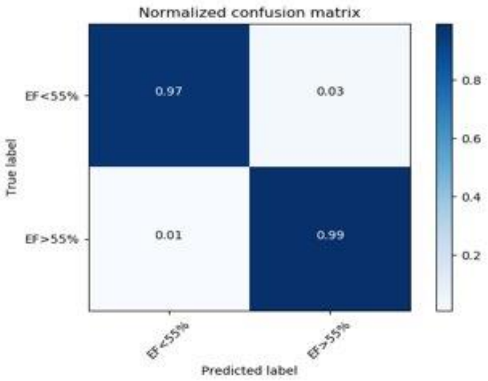

Decision Tree

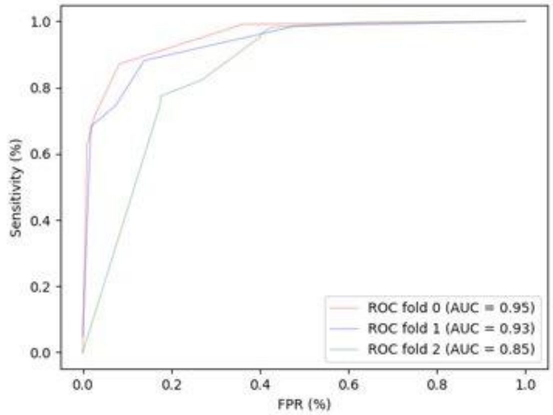

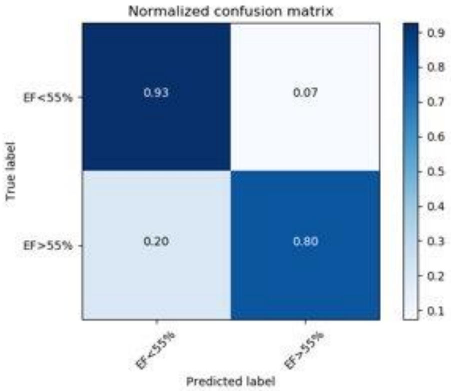

Figure S4. *NLP pipeline validation in subjects with EF data availability across structured and unstructured platforms. The aggregation was aimed at obtaining a comprehensive history of all ejection fraction assessments performed for a patient throughout his medical history.*

<table>
<tr><th>Deep learning model validation</th><th>Overall text extracted EF aggregation validation</th></tr>
<tr><td>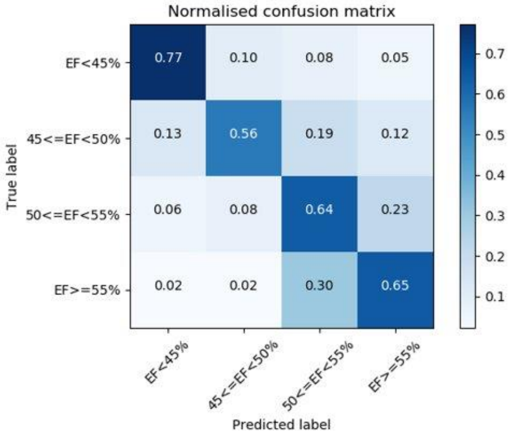
</td>
<td>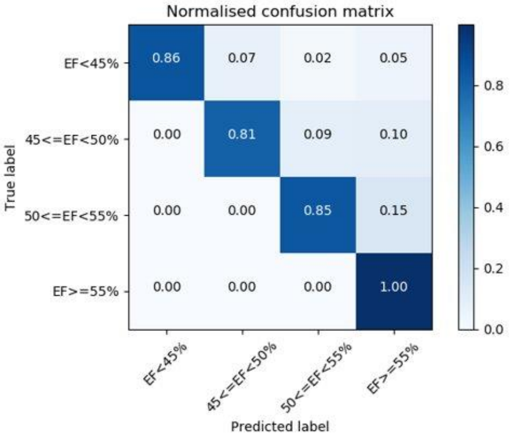
</td></tr>
</table>

Sec. IV: Data processing and signature training pipeline

Figure S5. *Data processing and machine learning pipeline for automated diagnosis of patients suffering from HFpEF of any disease stage.*

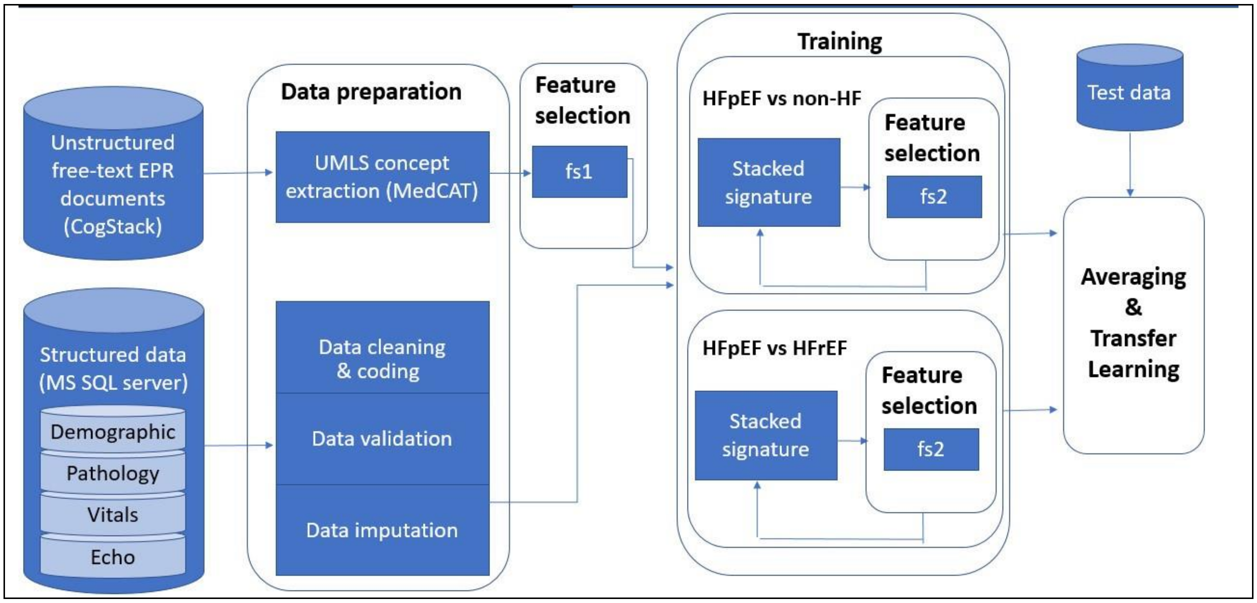

Sec. V: Echocardiographic measurements performed in KCH

Table S2. *Echocardiographic measurements and their unit of assessment used in the study. LV: Left ventricle, RV: Right ventricle, MV: Mitral valve, TV: Tricuspid valve, AV: aortic valve, HM: Heart model, BSA: Body surface are, PK EX: Peak exercise.*

LABEL	Unit of measurement	DESCRIPTION
AI MAX VEL	cm/sec	Aortic valve insufficiency max velocity
AO LVETC	sec	Aortic outflow
AO MEAN (PK EX)	cm/sec	Aortic outflow peak velocity at peak exercise
AO PEAK VEL (PK EX)	cm/sec	Aortic outflow mean velocity at peak exercise
AO V2 MAX	cm/sec	Aortic valve max velocity envelope of the distal flow
AO V2 MEAN	cm/sec	Mean velocity envelope of the distal flow in Aortic valve
AS MAX VEL	cm/sec	AS jet velocity max
AV PEAK VEL	cm/sec	Aortic valve peak velocity
AV VMAX-PR_PHL	cm/sec	Aortic valve systolic velocity prosthetic antegrade flow
E/E' AVERAGE	-	Mitral peak velocity of early filling (E) to early diastolic mitral annular velocity (E'), Average
E/E'_LAT	-	Mitral peak velocity of early filling (E) to early diastolic mitral annular velocity (E'), Lateral
E/E'_SEPT	-	Mitral peak velocity of early filling (E) to early diastolic mitral annular velocity (E'), Septal
EF (manual)	%	Ejection fraction
LVEF (automated HM)	%	Ejection fraction (heart model, automated)
IVR TIME	sec	Isovolumic relaxation time
LA AREA	cm^2	Left atrial area
LA DIMENSION	cm	Left atrial dimension
LA SYSTOLIC VOLUME (HM)	ml	Left atrial systolic volume
LA VOL (2D BIPLANE) INDEX BSA	ml/m^2	Left atrial volume normalised to body surface area
LA VOLUME (2D BIPLANE)	ml	Left atrial volume (2d biplane)
LA/AO	-	LA/AO
LV DP/DT	mmHg/s	Difference in the pressure through mitral valve inflow in time (isovolumic phase index of the LV systolic performance)
LV EDV (HM)	ml	LV end diastolic vol
LV ESV (HM)	ml	LV end systolic Vol
LV EXCURSION - AVG	cm	Mitral annular plane systolic excursion (MAPSE), average
LV EXCURSION - MAX	cm	Mitral annular plane systolic excursion (MAPSE), max
LV EXCURSION - MIN	cm	Mitral annular plane systolic excursion (MAPSE), min
LV EXCURSION - SD	cm	Mitral annular plane systolic excursion (MAPSE), std

LV MASS - END DIAS	g	LV mass - end diastolic
LV MASS - END SYS	g	LV mass - end systolic
LV MASS(AL) DIAS INDEXED	g/m^2	LV mass
LV MASS(C) SYS INDEXED	g/m^2	LV mass
LV PK 1	cm/sec	Max velocity of the flow proximal to the AV at stress echo (rest)
LV PK 2	cm/sec	Max velocity of the flow proximal to the AV at stress echo (low dose)
LV PK 3	cm/sec	Max velocity of the flow proximal to the AV at stress echo (peak)
LV STROKE VOLUME (HM)	ml	LV stroke volume
LV V1 MAX	cm/sec	Left ventricular max velocity proximal to AV
LV V1 MAX (PK EX)	mmHg	Left ventricular max pressure gradient at peak exercise
LV V1 MAX PG	mmHg	Left ventricular max pressure gradient
LV V1 MEAN	cm/sec	Left ventricular outflow tract mean velocity
LV V1 MEAN PG	mmHg	Left ventricular outflow tract mean velocity
LV V1 MEAN PG (PK EX)	mmHg	Left ventricular outflow tract mean velocity at peak exercise
LV V1 VTI	cm	Left ventricular outflow tract velocity time integral
LV V1 VTI (PK EX)	cm	Left ventricular outflow tract velocity time integral
LV VMAX 10MCG	mmHg	Max pressure gradient of flow proximal to AV
LV VMAX REST	mmHg	Max pressure gradient of flow proximal to AV at rest
LV VOL D (INDEXED BSA)	ml/m^2	LV volume diastolic normalised to body surface area
LV VOL S (INDEXED BSA)	ml/m^2	LV volume systolic normalised to body surface area
LV VTI 10MCG	cm	Left ventricular outflow tract velocity time integral
LV VTI 5MCG	cm	Left ventricular outflow tract velocity time integral
LV VTI REST	cm	Left ventricular outflow tract velocity time integral at rest
LVAD SAX MAX	cm^2	LV area at diastolic
LVAS SAX MV	cm^2	LV area at systole
LVD MASS/VOLUME	-	LV diastolic mass to volume
LVDIAS VOL 3D (INDEXED BSA)	ml/m^2	LV diastolic vol 3D
LVET	sec	Left ventricle Ejection time
LVIDD	cm	Left ventricular internal diameter end diastole
LVIDD (INDEXED BSA: CM/M^2)	-	Left ventricular internal diameter end diastole (normalised to BSA)
LVIDS	cm	Left ventricular internal diameter end systole
LVLD %DIFF	%	LV long axis difference ration at diastole
LVLD APICAL(4CH)	cm	LV long axis apical contour at diastole (4-chamber view)
LVLD EPI APICAL(4CH)	cm	LV epicardial long axis apical contour at diastole (4-chamber view)
LVLS %DIFF	%	LV long axis difference ration at systole
LVLS AP2	cm	LV long axis at systole (2-chamber view)
LVLS AP4	cm	LV long axis at systole (4-chamber view)

LVLS APICAL	cm	LV long axis apical contour at diastole (4-chamber view)
LVOT ACCEL SLOPE	cm/sec^2	LV outflow tract ACCEL slop (cm/s^2)
LVOT _AREA	cm^2	LV outflow tract area (automated possibly)
LVOT AREA	cm^2	LV outflow tract area (automated)
LVOT AREA(TRACED)	cm^2	LV outflow tract area (manual more accurate)
LVOT D (2D)	cm	LV outflow tract diameter
LVOT DIAM	cm	LV outflow tract diameter
LVPEP	sec	LV pre-ejection period
LVPWD	cm	Left ventricular posterior wall end diastole
LVPWS	cm	Left ventricular posterior wall end systole
LVS MAJOR	cm	LV major dimension at diastole
LVSYS VOL 3D (INDEXED BSA)	ml/m^2	LV systolic vol 3D indexed to BSA
MAX V	cm/sec	Max doppler velocity
MEAN V	cm/sec	Mean doppler velocity
MED PEAK S VEL	cm/sec	Peak s-wave velocity of the medial MV annulus tissue doppler spectrum
MITRAL R-R	sec	Mitral R-R time
MM R-R INT	sec	M-mode R-R interval
MR ALIAS VEL	cm/sec	Mitral regurgitation alias velocity
MR MAX VEL	cm/sec	Mitral regurgitation max velocity
MR MEAN VEL	cm/sec	Mitral regurgitation mean velocity
MV A DUR	sec	Mitral valve A-wave duration
MV A MAX VEL	cm/sec	Mitral valve A-wave max velocity
MV A MAX VEL (PK EX)	cm/sec	Mitral valve A-wave max velocity (peak exercise)
MV ACC TIME	sec	Mechanical ventilation acceleration time (MV: Mitral valve)
MV DEC SLOPE (PK EX)	cm/sec^2	Mechanical ventilation deceleration slope (MV: Mitral valve)
MV DEC TIME	sec	Mechanical ventilation deceleration time (MV: Mitral valve)
MV DEC TIME (PK EX)	sec	Mechanical ventilation deceleration time (peak exercise)
MV E MAX VEL	cm/sec	Mitral valve E-wave max velocity
MV E/A	-	Mitral valve E-wave/A-wave ratio
MV E-F SLOPE	cm/sec	Mitral valve E-F slope
MV P1/2T MAX VEL	cm/sec	Mitral valve P1/2T max velocity
MV V2 MAX	cm/sec	Mitral valve V2 (highest velocity at valve) max
MV V2 MAX (PK EX)	cm/sec	Mitral valve V2 (highest velocity at valve) max (peak exercise)
MV V2 MEAN	cm/sec	Mitral valve V2 (highest velocity at valve) mean (MV: Mitral valve)
PA ACC TIME	sec	Pulmonary velocity acceleration time
PA ACCEL TIME	sec	Pulmonary velocity acceleration time
PA V2 MAX	cm/sec	Pulmonary velocity max
PA V2 MEAN	cm/sec	Pulmonary velocity mean
PI MAX VEL	cm/sec	Pulmonary insufficiency max velocity

RAP systole	mmHg	Right atrial pressure at systole
RAP diastole	mmHg	Right atrial pressure at diastole
R-R TIME	sec	R-R time
RV V1 MAX	cm/sec	Right ventricle outflow tract max velocity
RV V1 MEAN	cm/sec	Right ventricle outflow tract mean velocity
TR MAX PG	mmHg	Tricuspid regurgitation max pressure gradient
TR MAX PG (PK EX)	mmHg	Tricuspid regurgitation max pressure gradient (peak exercise)
TR MAX VEL	cm/sec	Tricuspid regurgitation max velocity
TR MAX VEL (PK EX)	cm/sec	Tricuspid regurgitation max velocity (peak exercise)
TR VTI	cm	Tricuspid regurgitation velocity time integral
TV A MAX VEL	cm/sec	Tricuspid valve A-wave max velocity
TV DEC TIME	sec	Tricuspid valve deceleration time
TV E MAX VEL	cm/sec	Tricuspid valve E-wave max velocity
TV E/A	-	Tricuspid valve E/A ratio
TV P1/2T MAX VEL	cm/sec	Tricuspid valve P1/2T max velocity
TV V2 MAX	cm/sec	Tricuspid valve V2 max
TV V2 MEAN	cm/sec	Tricuspid valve V2 mean
VSD MAX VEL	cm/sec	Ventricular septal defect max velocity

Sec. VI: Performance metrics

F1-measure score = 2*precision*recall / (precision + recall)

Kappa score = (observed agreement – expected agreement) / (1 - expected agreement)

Expected agreement using the observed data is defined as the probabilities of each classifier model randomly predicting each category. This in effect for our binary classification task translates into:

Expected agreement = (n11*n12 + n21*n22)/N^2

Where N is the total number of samples (=269) and nik indicates the number of times the classifier i predicted category k (k in our study can take either of values, 0 indicative of no HFpEF and 1 indicative of HFpEF diagnosis.)

Sec. VII: Model adaptation

Figure S6. *Model adaptation optimization: different proportion of the test subject data were used to retune the model parameters for test-set usability and generalisation. Orange: AUROC performance metric, Blue: average precision (AP) performance metric.*

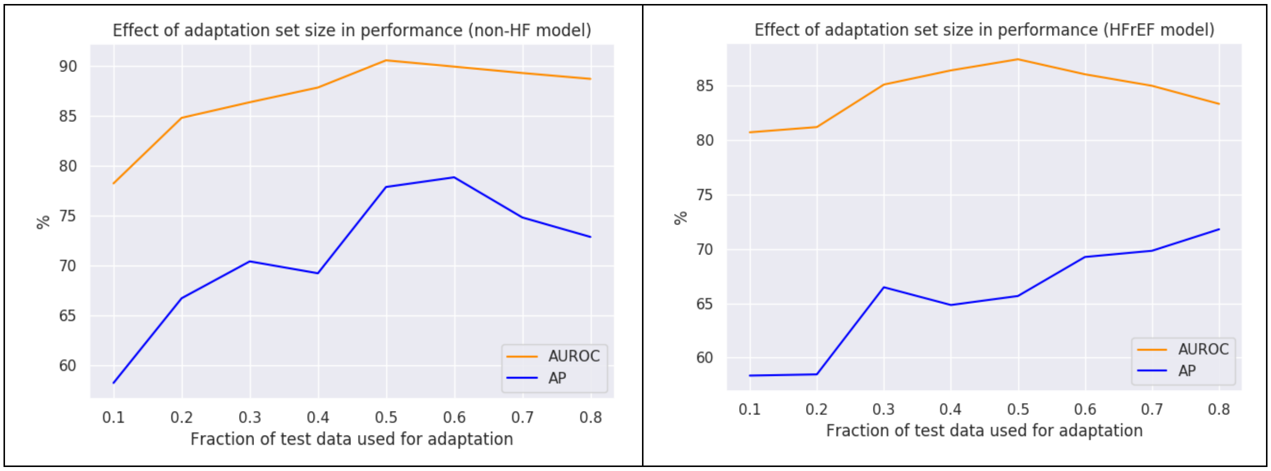

Sec. VIII: H2FPEF score computation

To compute the H2FPEF we have automated the data query for the six parameters that contribute in the score estimation. Missing values of the BMI were estimated using the patient's weight and height (BMI = weight/(height)**2). We have used the average value of the e/e' septal and lateral for filling pressure and the sum over the maximum tricuspid regurgitation pressure gradient and the right atrial pressure at systole[22] (where available) as the closets estimate of the pulmonary artery systolic pressure, proposed by [16]. The ICD10 code of I48*** has been used for ascribing prior diagnosis of atrial fibrillation. And the data from the UMLS drug hierarchy was queried to verify whether a prescribed medication is an antihypertensive drug - we used simple regex text cleaning, the UMLS repository and MedCAT medical text annotation tool to accomplish this. Following the linkage of these six data items for each patient, missing values on each item were replaced with the normal value of that parameter - replacements with abnormal values resulted in lower AUROC value for H2FPEF score - i.e. missing values of BMI were replaced by 29 kg/m2.

Measurements for each data item were then ascribed with scoring points following[16]. Figure S7 summarizes the scoring system proposed by[16].

Figure S7. *H2FPEF scoring system.*

	Clinical Variable	Values	Points
H_2	Heavy	Body mass index > 30 kg/m^2	2
	Hypertensive	2 or more antihypertensive medicines	1
F	Atrial Fibrillation	Paroxysmal or Persistent	3
P	Pulmonary Hypertension	Doppler Echocardiographic estimated Pulmonary Artery Systolic Pressure > 35 mmHg	1
E	Elder	Age > 60 years	1
F	Filling Pressure	Doppler Echocardiographic E/e' > 9	1
H_2FPEF score			Sum (0-9)

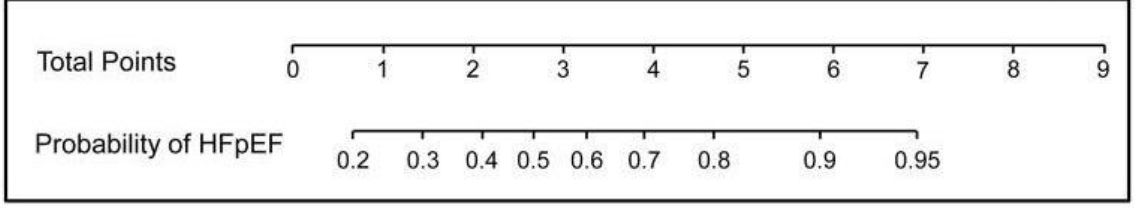

Sec IX: FN/FP analysis

Table S3: *Aggregate model False negative and False positive assessments. The highlighted lines are indicative of the FP cases where our Aggregate model assigned a HFpEF label incorrectly.*

Agg. Model Pred.	H2FPEF Score	Anno.	Clinical comments
1	3	0	Alternate diagnosis: COPD to explain breathlessness. Preserved LV function, and mildly dilated LA and LVH, but no other HF symptoms apart from dyspnoea
0	8	1	Does have a diagnosis of HFpEF
0	2	1	Does have a diagnosis of HFpEF - symptoms of heart failure, preserved EF on echo, positive BNP
0	2	1	Clinic letter from 2004 discussion diastolic dysfunction on echo, SOB and peripheral oedema. No BNP was ever reported.
1	6	0	Alternate diagnosis: AF causing pulmoary oedema, but this was precipitated by drugs in hospital
1	8	0	Two reports on the echocardiograms suggesting mildly or borderline reduced LV function
1	4	0	Alternate diagnosis: pulmonary emboli on a CT scan
1	4	0	Alternate diagnosis: SOB in context of pneumonia. Previous CABG for ischemic heart disease, but no consistent heart failure syndrome
1	5	0	Alternate diagnosis: end stage renal failure on dialysis. Episodes of fluid overload are not due to heart failure. Other episodes of breathlessness are in the context of infection.
1	6	0	Alternate diagnosis: severe aortic stenosis
1	4	0	Alternate diagnosis: breathlessness due to suspected infection
1	4	0	Alternate diagnosis: Breathlessness due to chest infection / reaction to antibiotic
1	3	0	Alternate diagnosis: Ischaemic heart disease
1	3	0	Alternate diagnosis: pulmonary hypertension due to pulmonary haemorrhage
0	4	1	Sickle cell patient. Does have some echo features of HFpEF but symptoms in the context of leukemia/pericardial effusion/presumed PE, therefore always an alternate diagnosis.
1	5	0	Alternate diagnosis: Severe tricuscpid regurgitation due to a pacemaker lead problem
1	4	0	Alternate diagnosis: diabetic patient with reduced EF on echo
1	5	0	Alternate diagnosis: Hypertrophic cardiomyopathy
1	6	0	Alternate diagnosis: Severe aortic stenosis
1	9	0	Alternate diagnosis: Right ventricular dysfunction

1	2	0	Alternate diagnosis: ischemic heart disease and moderate aortic regurgitation, lung disease causing breathlessness
1	7	0	Alternate diagnosis: Severe COPD with normal BNP
1	4	0	Alternate diagnosis: Aortic valve replacement
1	6	0	Alternate diagnosis: ischemic heart disease and pulmonary fibrosis
1	4	0	No consistent history of heart failure
1	8	0	Alternate diagnosis: Aortic valve disease
1	8	0	Alternate diagnosis: AF, Symptoms of oedema improved post-cardioversion
1	3	0	Alternate diagnosis: Severe lung disease
1	4	0	Alternate diagnosis: Right ventricular dysfunction
1	8	0	Alternate diagnosis: Ischaemic heart disease requiring multiple stents
0	1	1	Does have HFpEF Breathlessness, preserved LV function, positive BNP. On the initial echo in September 2019 the TR was only mild-moderate, so this is enough to make the diagnosis. Subsequently her TR worsened to severe
1	6	0	Alternate diagnosis -> pulmonary fibrosis
1	5	0	Alternate diagnosis: Hypertrophic cardiomyopathy
